# Reliable but multi-dimensional cognitive demand in operating partially automated vehicles: implications for real-world automation research

**DOI:** 10.1186/s41235-024-00591-5

**Published:** 2024-09-11

**Authors:** Monika Lohani, Joel M. Cooper, Amy S. McDonnell, Gus G. Erickson, Trent G. Simmons, Amanda E. Carriero, Kaedyn W. Crabtree, David L. Strayer

**Affiliations:** 1 Department of Psychology, University of Utah, 380 S 1530 E BEHS 1003, Salt Lake City, UT 84112 USA; 2 Red Scientific Inc., Salt Lake City, UT USA

**Keywords:** Cognitive demand, Automation, Psychophysiology, Performance, Real-world

## Abstract

The reliability of cognitive demand measures in controlled laboratory settings is well-documented; however, limited research has directly established their stability under real-life and high-stakes conditions, such as operating automated technology on actual highways. Partially automated vehicles have advanced to become an everyday mode of transportation, and research on driving these advanced vehicles requires reliable tools for evaluating the cognitive demand on motorists to sustain optimal engagement in the driving process. This study examined the reliability of five cognitive demand measures, while participants operated partially automated vehicles on real roads across four occasions. Seventy-one participants (aged 18–64 years) drove on actual highways while their heart rate, heart rate variability, electroencephalogram (EEG) alpha power, and behavioral performance on the Detection Response Task were measured simultaneously. Findings revealed that EEG alpha power had excellent test–retest reliability, heart rate and its variability were good, and Detection Response Task reaction time and hit-rate had moderate reliabilities. Thus, the current study addresses concerns regarding the reliability of these measures in assessing cognitive demand in real-world automation research, as acceptable test–retest reliabilities were found across all measures for drivers across occasions. Despite the high reliability of each measure, low intercorrelations among measures were observed, and internal consistency was better when cognitive demand was estimated as a multi-factorial construct. This suggests that they tap into different aspects of cognitive demand while operating automation in real life. The findings highlight that a combination of psychophysiological and behavioral methods can reliably capture multi-faceted cognitive demand in real-world automation research.

## Significance statement

Several laboratory-based measures are highly stable in controlled settings, but how do they perform in less controlled and arguably more “noisy” real-world conditions? We examined the reliability of five commonly utilized laboratory-based measures of cognitive demand that may be good candidates for real-world assessments while participants operated four partially automated vehicles on actual highways. The main focus of this research was to examine the reliability of a combination of behavioral, peripheral, and central nervous system-based measures while the vehicles were being driven under partial automation to make inferences about the applicability of these measures in automation research in real-life settings. Good to excellent reliabilities were found for all measures, providing confidence that they can be successfully adopted to measure cognitive demand while motorists operated automation on actual roads.

## Introduction

In 2024, about 54 million vehicles with some form of automation are expected to be driven on real roads, making partially automated vehicles an everyday mode of transportation (Statista, 2023). These forecasts also highlight the need for objective and reliable ways to measure motorists’ cognitive states while driving partially automated vehicles in real-world conditions. While many physiological and behavioral measures of cognition can be reliably measured in real time in controlled laboratory settings, limited research has tested the reliability of these measures under real-world conditions (Lohani et al., 2019). The current work aimed to address this knowledge gap and extend the applicability of reliable measures to real-world automation research.

The Society of Automotive Engineers (SAE, 2016) identifies automation along a spectrum varying from none or manual (Level 0) to full automation (Level 5), which can operate without any human involvement. While Level 5 is still unavailable to consumers for purchase for several years to come (Bazilinskyy et al., 2019), partially automated vehicles (that still require human involvement in the driving process) are readily available and are becoming quite popular. The most prevalent advanced partial automation available is Level 2, which allows the automation of lateral (e.g., steering) and longitudinal (e.g., braking) control of the vehicle at the same time (SAE, 2016). Although commercial automation technology can perform several key driving tasks, it is still quite susceptible to error, making it necessary for motorists to be always engaged in the driving process. However, human drivers’ distractions and misuse of automation features have been identified as a few of the major reasons for partial automation vehicle collisions (e.g., National Transportation Safety Board, 2020). Such findings suggest that having more objective ways to assess cognitive states in partially automated vehicles would be valuable in identifying sub-optimal levels of human cognitive engagement in the driving task.

At the same time, an important purpose of automation is to lower task demands imposed on humans, thereby maintaining task performance for long durations, reducing human error, and improving safety (Scerbo, 2007). Thus, having reliable ways of measuring task demands imposed on motorists operating partially automated vehicles in near-real-time, particularly cognitive demand, would help assess and manage them effectively. Ideally, it would also help to have measures that are minimally disruptive to the primary driving task, allowing for a more accurate assessment. It would also, therefore, help to know how different measures of cognitive demand tap into the same construct because multiple measures for the same construct are widely utilized. This would help draw comparisons across measures and studies on cognitive demand imposed while operating automation.

The driving task recruits several systems, such as visual perception, motor, attention, planning, and memory (e.g., Bergen et al., 2013; Engström et al., 2005; Lohani et al., 2019; Strayer, 2015). Given that several cognitive demand measures are available to be potentially measured at the same time, domain-general versus domain-specific perspectives are a useful way to understand how different measures are tapping into one global or multi-faceted cognitive demand construct. If multiple cognitive measures tap into the domain-general resource for the driving task (Kahneman, 1973; Navon & Gopher, 1979), there would be a greater overlap between the measures. In contrast, if the measures were tapping into separable systems due to the multi-faceted nature of the driving task and aligned with domain-specific theory (Wickens, 1984), there would be a negligible to small overlap between the measures. To identify relevant measures, in a review of potential measures for assessing cognition during real-world driving, Lohani et al. (2019) have argued that a critical starting point is to select measures that can reliably measure cognition in controlled laboratory settings and then adapt them to naturalistic environments. Further, this review found some peripheral (cardiovascular) and central nervous system-based (electroencephalogram; EEG) measures as good candidates for near real-time and real-world assessment of drivers’ cognitive states.

One of the most promising peripheral nervous system-based measures is *heart rate* (Berntson et al., 2007), which is the count of heartbeats in a minute (also called beats per minute; BPM). It represents physiological arousal due to cognitive demand in automation research (Hidalgo-Muñoz et al., 2018; Lenneman & Backs, 2009; Mehler et al., 2012; Ruscio et al., 2017). A higher cognitive demand (such as workload) while driving on-road is systematically linked to increased heart rate (e.g., Reimer et al., 2009). A less common cardiovascular measure is *heart rate variability* (HRV), which captures variability in adjacent heartbeats and is associated with cognitive demand during driving-related and goal-directed behavior (Heine et al., 2017; Hidalgo-Muñoz et al., 2018; Lee et al., 2007; Tozman et al., 2015; Zhao et al., 2012). A common heart rate variability measure in automation research is a time domain-based parameter called the *Root-Mean-Square of Successive Differences* (RMSSD) in normal heartbeats, which measures the temporal variability in adjacent heartbeats. Decreases in RMSSD values while driving represent increases in cognitive demand, such as workload (Mehler et al., 2011).

Furthermore, a central nervous system-based method is the electroencephalogram (EEG), which can be used to examine the EEG alpha power. To provide some background information, the EEG records the brain’s electrical activity in voltages through electrodes placed on specific locations of a person’s scalp (Nunez & Srinivasan, 2006). These electrical signals have a high temporal resolution (in milliseconds). Existing transformations (such as the fast Fourier transformation) can convert the time domain EEG signal (in milliseconds) into the frequency domain (in Hz), thereby creating a spectrum of frequencies, allowing researchers to study specific frequency bands that have meaningful interpretations for cognitive activity. The *EEG alpha power* is the power in the 8–12 Hz spectral frequency band and is one of the most well-studied and prominent oscillatory components in the EEG literature (e.g., Donoghue et al., 2022; Klimesch, 2012). Moreover, alpha is clearly distinguished from background neural activity in the EEG frequency spectrogram (Donoghue et al., 2022), observed as a clear peak in the 8–12 Hz frequency range that is maximal over posterior electrodes. EEG alpha power reflects the functional inhibition of sensory input (Jensen & Nazaheri, 2010) and, as such, variations in cognitive workload (Käthner et al., 2014). Furthermore, in the driving context, increases in cognitive demand are well-documented to decrease EEG alpha power (Borghini et al., 2014; Käthner et al., 2014), whereas more relaxed conditions increase it (Zander et al., 2017). Thus, EEG alpha power is a valid measure of cognitive workload in driving research (for more details, see Lohani et al., 2019).

In addition to these physiological measures, a standardized behavioral measure commonly adopted in driving research is the *Detection Response Task* (DRT; ISO 17488 guidelines, 2016). It involves outfitting a motorist with a vibrating device on their arm and instructing them to manually respond while operating the vehicle as soon as the device vibrates. The reaction time and accuracy performance on the Detection Response Task are examined with slower and inaccurate responses representing higher cognitive workload. This vibrotactile detection measure has been specifically designed and standardized to assess cognitive workload that is sensitive to driving-related tasks (ISO 17488 guidelines, 2016) without significant additional cost (Castro et al., 2019). It has also been successfully adopted in partial automation research (e.g., Lohani et al., 2020, 2021). Slowing down and increased errors on the Detection Response Task represent increases in driving-related cognitive workload (Young et al., 2013). To summarize, these five measures discussed above are sensitive to various driving-associated cognitive demands, which makes them suitable for measuring cognitive demand during automated driving conditions.

### The current study

Several laboratory-based measures have high reliability in controlled settings, but how do they perform in less controlled and arguably more “noisy” real-world conditions? We examined the reliability of five cognitive demand measures while participants operated partially automated vehicles across four occasions in real-world driving conditions. Participants drove on actual highways on four different occasions while their heart rate (measured in BPM), heart rate variability (measured using RMSSD), EEG alpha power, Detection Response Task reaction time, and Detection Response Task hit-rate data were collected simultaneously. The main focus of this research was to examine these relations while the vehicles were being driven under partial automation to make inferences about the applicability of these measures in automation research in real-life settings. However, for reference, we also report data while the same vehicles were being operated in manual mode.

Two research questions were examined: First, how stable are measures of cognitive demand when assessed on multiple occasions in real-world conditions? In order to examine the reliability of a measure over time, test–retest reliability using the intraclass correlation (ICC) was calculated for each measure separately (Weir, 2005). Specifically, ICC1 (McGraw & Wong, 1996; Shrout & Fleiss, 1979) was assessed as this coefficient captures the reliability of a measure across occasions. A similar approach has been adopted in comparable work examining the test–retest stability of measures to capture a cognitive workload construct in listening research (Alhanbali et al., 2019). As these cognitive measures have been shown to be reliable in laboratory settings, we argued that successful adaptation to real-world driving would require similarly high measures of test–retest reliability on the road to what has been observed in the laboratory under manual and partially automated driving.^1^

Second, how do these different measures of cognitive demand intercorrelate with one another? These measures may all tap into a general construct of cognitive demand that is at play while operating automation on real roads, leading to high intercorrelations and highly reliable coefficients. Alternatively, a multi-faceted construct may better capture the construct of cognitive workload, which would be reflected by lower correlations between measures and a better fit (i.e., higher internal consistency) when comparing a multi-factorial factor structure representing more than one latent variable to a general factor saturation. A few psychometric coefficients that capture how well the measures tap into a similar construct were evaluated, including omega (McDonald, 1999; see data analysis plan). A simultaneous assessment of multiple measures enabled an examination of overlap across various cognitive demand measures and whether they tap into the same construct. Given that a limited understanding exists across different cognitive demand measures in real-world settings, we explored whether the multiple measures tap into a general versus multi-faceted construct of cognitive workload.

## Method

### Participants

Seventy-one participants (25 females) between the ages of 21 and 64 (*M*_age_ = 40.32 years, *SD* = 13.37) took part in this study. This age range was based on guidelines for automation testing (NHTSA, 2013). Eligibility criteria included no neurological or cardiovascular health issues, no self-reported experience with partially automated vehicles, having a valid driver’s license, and a good driving record (e.g., no at-fault accidents in the previous two years). The University of Utah’s Division of Risk Management confirmed the driving records. All screened participants also completed the Division of Risk Management’s online defensive driving course.

### Measures

#### Heart rate (BPM) and its variability (RMSSD)

Heart rate data were sampled (2000 Hz) via an electrocardiogram (ECG) system and AcqKnowledge software (Biopac System Inc.). Data were processed using the recommended guidelines for cardiovascular data (e.g., Berntson et al., 2007; Task Force of the European Society of Cardiology, 1996). The ECG signal was first band-pass filtered (1–35 Hz), followed by detecting R-peaks via AcqKnowledge software. Due to the real-world nature of the driving task, potential artifacts (such as those from the driving task or sneezes, etc.) are expected to be present as noise. Thus, following recommendations to remove any such artifacts, all data were visually inspected and corrected for proper detection of R-peaks. After completing data processing, heart rate and heart rate variability (RMSSD) were calculated.

#### EEG alpha power

A gel-based system (Biopac Systems Inc.) was used to collect EEG data sampled (2000 Hz) from the midline parietal site (Pz^2^; Jasper, 1958). MATLAB and the EEGLAB toolbox were utilized to process data using best practices (Delorme & Makeig, 2004). These included a 0.1–30 Hz band-pass filter followed by one-second epochs using a Hanning window and removal of blinks and eye movement artifacts using Gratton’s regression-based correction protocol (Gratton et al., 1983). A moving window approach was used to further check for remaining artifacts by rejecting any epochs with flatlines or peak-to-peak activity higher than 200 μV (Lopez-Calderon & Luck, 2014). After removing possible artifacts, a fast Fourier transform was utilized to calculate the average alpha power in the 8–12 Hz frequency band (Cohen, 2014).

#### Detection response task

Performance on the Detection Response Task (ISO17488, 2016) was used to capture the cognitive demand behaviorally. The standardized protocol was followed by placing a small motor that vibrates every 3–5 s on participants’ left biceps (to avoid disrupting ECG signals, as previously done; Lohani et al., 2020). Participants’ goal was to respond to the vibration onset as quickly as possible while driving (with priority always given to safe driving) by pressing a microswitch attached to the left hand’s index or middle finger (ISO17488, 2016). The equipment and software were provided by Red Scientific Inc., which presented the stimuli and collected the responses. Vibration was presented only for a second and then turned off. If the participant had to miss a response due to driving reasons, it was excluded from the analysis. An increase in driving-related cognitive workload is associated with an increase in reaction time and worse hit-rate performance on the Detection Response Task (Young et al., 2013).

### Procedure

All procedures were approved by the Institutional Review Board of the University of Utah. During the first visit, participants read and signed the consent form. Four partially automated vehicles (Cadillac CT6, Nissan Rogue, Tesla Model 3, and Volvo XC90) were tested, with a separate visit^3^ for each (counterbalanced across participants). Physiological equipment was placed on participants in the lab. Next, the Detection Response Task equipment was set up, and all signals were checked in the parking lot. Participants were trained on operating the car’s automation features. The study was conducted within typical work hours, 9 am to 5 pm, over weekdays. Given the naturalistic conditions of driving on real highways, some variability^4^ in traffic patterns is expected. Considering this, a large sample size was collected with the data collected within work hours, and the order was counterbalanced for highways across participants to increase generalizability to highway driving. Participants got to drive on a practice route before they started driving on pre-determined testing routes for all participants. On the same highways, participants drove on partial automation one way and the manual mode the other way, which took at least 18 min each way and was counterbalanced across conditions and people. Comparable traffic was found in both directions. If participants took slightly longer, only 18 min of data were included for consistency across participants. A research assistant was always present in the vehicle to monitor safe driving practices and the quality of data collection in real time.

### Data analysis plan

In order to address the first question about the test–retest reliability of each measure, intraclass correlations (ICCs) were estimated, which can range between 0 (no reliability) and 1 (perfect reliability; Weir, 2005). In particular, an *ICC1* (McGraw & Wong, 1996; Shrout & Fleiss, 1979) estimates reliability for a single score from each subject across four occasions and is calculated by fitting a 1-way random effects model and estimating ICC via a ratio of the estimated variance components. Based on work by Weir (2005), a random effects model was selected because the partially automated vehicles were a sample of all possible options, thereby making the vehicles a random effect and allowing the findings to be generalized to other automation vehicle types as well. Note that vehicle-specific differences were not examined because no effect of vehicle type was found for the measures of cognitive demand in on-road driving (Lohani et al., 2021; McDonnell et al., 2023). In accordance with previous recommendations (McGraw & Wong, 1996; Shrout & Fleiss, 1979; Weir, 2005), while calculating intraclass correlation, a linear mixed model was used, and if the model did not converge, an analysis of variance approach was adopted as an alternative (Weir, 2005).

Based on guidelines (Revell & Condon, 2019; Shrout & Lane, 2012), to test the second question regarding the overall intercorrelation of cognitive demand measures, four coefficients were examined: (1) *omega hierarchical* (*ω*_*h*_; McDonald, 1999) is an internal consistency coefficient based on a general factor, (2) *omega total* (ω_t_; McDonald, 1999) is a coefficient that measures reliability assuming a multi-factor structure across measures; (3) *R1R* (Shrout & Lane, 2012) measures the generalizability across all items (based on a random time effect); (4) *Cronbach’s alpha* (α; Cronbach, 1951) is the most commonly reported coefficients of generalizability.^5^ These coefficients would provide information to evaluate the generalizability of the multiple measures of cognitive demand that were assessed simultaneously across four occasions. All analyses were conducted in RStudio (2016).

## Results

### Test–retest reliabilities for each measure

Table 1 presents the intraclass correlations for each of the five cognitive demand measures and their 95% confidence intervals. Based on the previous classifications for intraclass correlation coefficients (Koo & Li, 2016), excellent test–retest reliability was found for EEG alpha power, followed by good reliability for heart rate (in BPM) and moderate reliability for heart rate variability (in RMSSD) measure. Moderate reliability was also found for the Detection Response Task’s reaction time and hit-rate measures. A comparison of intraclass correlation confidence intervals for partial automation and manual modes showed overlap, suggesting comparable coefficients for both.

**Table 1:** ICC1s (McGraw & Wong, 1996; Shrout & Fleiss, 1979) for each measure are plotted during partial automation and manual mode driving

Measures	ICC1 95% CI [LL, UL]	
	Partial automation	Manual
Heart rate (BPM)	0.7975 [0.7385, 0.8492]	0.8722 [0.7818, 0.9366]
Heart rate variability (RMSSD)	0.7196 [0.6460, 0.7875]	0.7669 [0.7009, 0.8257]
EEG alpha power	0.9581 [0.9233, 0.9805]	0.9398 [0.9191, 0.9567]
DRT reaction time	0.6947 [0.5223, 0.8403]	0.7469 [0.6768, 0.8098]
DRT Hit-rate	0.6170 [0.5274, 0.7027]	0.6273 [0.5383, 0.7120]

Heart rate is measured in beats per minute (BPM). Heart rate variability is measured by a time domain-based parameter called the root-mean-square of successive differences (RMSSD). DRT is the behavioral performance on the Detection Response Task

95% confidence intervals (CI) are presented in brackets

### Internal consistency across measures

Table 2 presents the within and between-person level correlations across all measures. Although there were some significant correlations, such as associations between cardiovascular measures and behavioral measures, there was a notable lack of correlations between cardiovascular and EEG measures at both the within- and between-person levels, and generally, correlation coefficients were low across measures derived from different measurement tools.

**Table 2:** For all the measures, within-person level correlations across the four occasions are reported above the middle blank line, and between-person correlations are reported below it for a) partial automation mode and b) manual driving mode

	Heart rate (BPM)	Heart rate variability (RMSSD)	EEG alpha power	DRT reaction time	DRT hit-rate
a) Partial Automation					
Heart rate (BPM)		− 0.66***	0.09	− 0.01	0.11
Heart rate variability (RMSSD)	− 0.4***		− 0.04	0.07*	− 0.04
EEG alpha power	0.03	− 0.18		0.19*	− 0.17*
DRT Reaction Time	− 0.04	− 0.21	0.26*		− 0.33***
DRT Hit-rate	0.15	0.38**	− 0.3*	− 0.63***	1
b) Manual mode					
Heart rate (BPM)		− 0.75***	0.01	− 0.09	0.11
Hear rate variability (RMSSD)	− 0.47***		0.01	0.05	− 0.11
EEG alpha power	− 0.02	− 0.1		0.13	− 0.16*
DRT Reaction Time	0.01	− 0.23	0.28*		− 0.27**
DRT Hit-rate	0.09	0.27*	− 0.26*	− 0.66	

*is *p* < 0.05; ** is *p* < 0.01; *** is *p* < 0.001. Heart rate is measured in beats per minute (BPM). Heart rate variability is measured by a time domain-based parameter called the root-mean-square of successive differences (RMSSD). DRT is the behavioral performance on the Detection Response Task

The results of internal factor(s)-based coefficients are reported in Table 3. The *omega hierarchical* (*ω*_*h*_; McDonald, 1999) coefficient has been proposed as the best estimate of a general factor (Revelle & Zinbarg, 2009; Zinbarg et al., 2006). It indicated low reliability based on a general factor (or a single latent variable solution) for cognitive demand across all the measures. In contrast, the *omega total* (*ω*_t_; McDonald, 1999, which allows for a multi-factor solution and is based on all factors) was indicative of higher internal consistency, supporting cognitive demand as a multi-factor construct specifically in the context of automation and manual driving on the road. The R1R coefficient of generalizability across items utilizing random time effect (Shrout & Lane, 2012) had similar estimates as the omega total.

**Table 3:** Intercorrelation coefficients of cognitive demand measures are presented for partial automation and manual modes

	Partial automation	Manual
Omega hierarchical (*ω*_*h*_)^+^	0.36	0.34
Omega total (*ω*_*t*_)^#^	0.67	0.73
R1R	0.68	0.68
Cronbach’s alpha	0.58	0.37

^+^Omega is designed for internal consistency at an occasion (Revelle & Condon, 2019); thus, in order to calculate a single coefficient analogous to other reliability coefficients, an average *ω*_*h*_ was calculated across four occasions of partial automation mode (0.3, 0.3, 0.52, 0.33) and manual mode (0.42, 0.24, 0.29, 0.4)

^#^Similar to *ω*_*h*,_ for interpretation, average *ω*_*t*_ values are reported by averaging across occasions for partial automation mode (0.66, 0.78, 0.85, 0.37) and manual mode (0.78, 0.82, 0.86, 0.45)

## Discussion

Partially automated vehicles show growing promise for safer driving as long as the motorists are cognitively engaged in the driving process and ready to take over when automation fails (NHTSA, 2013; SAE, 2016). However, motorists can inaccurately assess their cognitive states (Schmidt et al., 2009) and misuse automation altogether, thereby creating unsafe conditions for operating partially automated vehicles on actual highways. Thus, objective measures are needed to evaluate optimal levels of cognitive demand experienced by motorists during real-world driving tasks, such as driving on actual highways, which are less controllable and more demanding than traditional laboratory tasks. The current study was the largest effort to examine the reliability of using a combination of peripheral and central nervous system-based and behavioral measures of cognitive demand based on a representative sample of motorists who operated partial automation vehicles on real roads.

An examination of the stability of laboratory-based measures under uncontrollable highway driving conditions revealed that EEG alpha power had excellent reliability. Similarly, cardiovascular measures of heart rate and its variability (RMSSD) had good reliability, and Detection Response Task performance measures had moderate reliabilities. Thus, the current study addresses concerns regarding the reliability of these measures in real-world automation research, as acceptable test–retest reliabilities were found across all measures for drivers across occasions. While these findings should be replicated and extended to be generalized to other automation research (e.g., public transportation, aviation, and military), they imply that the psychometric properties were remarkably good and that these measures may be successfully adapted to applied automation research.

Despite the acceptable temporal stability of each measure, we observed low correlations between each measure, suggesting that they tap into different aspects of cognitive demand during driving instead of a general cognitive demand construct. As evident across multiple measures of internal consistency (such as *ω*_*h*_), the generalizability was low for a general cognitive demand factor (Kahneman, 1973). However, when cognitive demand was estimated as a multi-factorial construct (Wickens, 1980, 1984), much better internal consistency was found across measures assessed during partial automation and manual driving in real-world contexts. This implies that it is better to conceptualize cognitive demand while operating automation as a multi-dimensional construct than to force a general latent construct. These findings are consistent with past work that has found support for multi-factor solutions for related constructs in real-world conditions, such as listening effort (Alhanbali et al., 2019) and workload (Matthews et al., 2015). Low correlations across measures and support for a multi-dimensional construct also highlight the importance of utilizing a combination of psychophysiological and behavioral methods to capture multi-faceted cognitive demand in applied settings (Lohani et al., 2019). These findings support a growing literature highlighting the applicability of multi-modal measures to studies of real-world automation research (de Waard & Lewis-Evans, 2014; Lohani et al., 2019).

Some limitations and future directions of the study should be considered. First, it was not an extensive evaluation of all cognitive demand measures, and future work should include additional measures to expand on the current work (e.g., pupillometry, thermal and optical imaging). Second, even though the study was conducted on highways, a research assistant was sitting in the car to ensure safe driving procedures, which could have influenced operators’ driving behavior. Third, it would also help to specifically target high-stress scenarios while operating partial automation, such as automation failures, which would allow testing the sensitivity of cognitive demand measures. Although these scenarios could not be manipulated for ethical reasons, but longitudinal data collection could capture these instances, as they naturally happen in real-world settings. Fourth, we did not assess how long durations of data collection are, when factors like vigilance or fatigue may start varying and may impact measures like error rates performance on Detection Response Task (Taylor-Phillips et al., 2024), and future work should examine this in real-world settings. Finally, this study examined Level 2 partially automated vehicles; future work is needed to extend this work to highly automated vehicles (Levels 3–5) in real-world settings.

## Conclusion

We examined the reliability of a combination of behavioral, peripheral, and central nervous system-based measures for a near real-time assessment of drivers’ cognitive demand. Good to excellent reliabilities were found for all measures, providing confidence in their successful adoption in real-world automation research. While a general factor solution fit the data poorly, a multi-factorial model yielded improved measures of reliability, suggesting that these measures tap into a multi-dimensional cognitive demand construct while operating automation on real roads.

## Data Availability

The datasets used and/or analyzed during the current study are available from the corresponding author upon reasonable request.

## Footnotes

1 Given that these measures are being used to examine manual and partial automation-related cognitive workload, we wanted to test the reliability separately under separable driving conditions.

2 In accordance with best practices, a ground electrode was placed on the center of the forehead, the reference electrode was placed on the right mastoid, and the electrooculographic activity was recorded from two electrodes above and below the right eye (Luck, 2014).

3 On the day of the study, participants should have slept a min of 6 h and had a 0% blood alcohol level as verified by a breathalyzer.

4 In past analyses, no significant differences within and between conditions were found for cognitive demands (see Lohani et al., 2021).

5 However, it has been strongly criticized and discouraged (Revell, 2013) as a measure of stability of a test, reliability, or internal consistency due to its lack of reflection of the structure of the test, lack of auto-correlation due to short-term state consistency equivalence (such deviations will lead to α underestimating reliability; Teo & Fan, 2013).

## References

[CR1] Alhanbali, S., Dawes, P., Millman, R. E., & Munro, K. J. (2019). Measures of listening effort are multi-dimensional. *Ear and Hearing,**40*(5), 1084. 30747742 10.1097/AUD.0000000000000697 PMC7664710

[CR2] Bazilinskyy, P., Kyriakidis, M., Dodou, D., & de Winter, J. (2019). When will most cars be able to drive fully automatically? Projections of 18,970 survey respondents. *Transportation Research Part F: Traffic Psychology and Behaviour,**64*, 184–195. 10.1016/j.trf.2019.05.008

[CR3] Bergen, B., Medeiros-Ward, N., Wheeler, K., Drews, F., & Strayer, D. L. (2013). The crosstalk hypothesis: Language interferes with driving because of modality-specific mental simulation. *Journal of Experimental Psychology. General,**142*, 119–130. 22612769 10.1037/a0028428

[CR4] Berntson, G. G., Quigley, K. S., & Lozano, D. (2007). Cardiovascular psychophysiology. *Handbook of Psychophysiology,**3*, 182–210. 10.1017/CBO9780511546396.008 10.1017/CBO9780511546396.008

[CR5] Borghini, G., Astolfi, L., Vecchiato, G., Mattia, D., & Babiloni, F. (2014). Measuring neurophysiological signals in aircraft pilots and car drivers for the assessment of mental workload, fatigue and drowsiness. *Neuroscience and Biobehavioral Reviews,**44*, 58–75. 10.1016/j.neubiorev.2012.10.003 23116991 10.1016/j.neubiorev.2012.10.003

[CR6] Castro, S. C., Strayer, D. L., Matzke, D., & Heathcote, A. (2019). Cognitive workload measurement and modeling under divided attention. *Journal of Experimental Psychology: Human Perception and Performance,**45*(6), 826. 30998070 10.1037/xhp0000638

[CR7] Cohen, M. X. (2014). Fluctuations in oscillation frequency control spike timing and coordinate neural networks. *Journal of Neuroscience,**34*(27), 8988–8998. 24990919 10.1523/JNEUROSCI.0261-14.2014 PMC6608248

[CR8] Cronbach, L. J. (1951). Coefficient alpha and the internal structure of tests. *Psychometrika,**16*, 297–334. 10.1007/BF02310555 10.1007/BF02310555

[CR9] Delorme, A., & Makeig, S. (2004). EEGLAB: An open source toolbox for analysis of single-trial EEG dynamics including independent component analysis. *Journal of Neuroscience Methods,**134*, 9–21. 10.1016/j.jneumeth.2003.10.009 15102499 10.1016/j.jneumeth.2003.10.009

[CR10] de Waard, D., & Lewis-Evans, B. (2014). Self-report scales alone cannot capture mental workload: A reply to De Winter, Controversy in human factors constructs and the explosive use of the NASA TLX: A measurement perspective. *Cognition, Technology & Work,**16*, 303–305. 10.1007/s10111-014-0277-z

[CR11] Donoghue, T., Schaworonkow, N., & Voytek, B. (2022). Methodological considerations for studying neural oscillations. *European Journal of Neuroscience,**55*(11–12), 3502–3527. 34268825 10.1111/ejn.15361 PMC8761223

[CR12] Engström, J., Johansson, E., & Östlund, J. (2005). Effects of visual and cognitive load in real and simulated motorway driving. *Transportation Research Part F: Traffic Psychology and Behaviour,**8*(2), 97–120. 10.1016/j.trf.2005.04.012

[CR13] Gratton, G., Coles, M. G., & Donchin, E. (1983). A new method for off-line removal of ocular artifact. *Electroencephalography and Clinical Neurophysiology,**55*, 468–484. 10.1016/0013-4694(83)90135-9 6187540 10.1016/0013-4694(83)90135-9

[CR14] Heine, T., Lenis, G., Reichensperger, P., Beran, T., Doessel, O., & Deml, B. (2017). Electrocardiographic features for the measurement of drivers’ mental workload. *Applied Ergonomics,**61*, 31–43. 10.1016/j.apergo.2016.12.015 28237018 10.1016/j.apergo.2016.12.015

[CR15] Hidalgo-Muñoz, A. R., Béquet, A. J., Astier-Juvenon, M., Pépin, G., Fort, A., Jallais, C., et al. (2018). Respiration and heart rate modulation due to competing cognitive tasks while driving. *Frontiers in Human Neuroscience,**12*, 525. 10.3389/fnhum.2018.00525 30687043 10.3389/fnhum.2018.00525 PMC6338053

[CR16] International Organization for Standardization (2016). ISO/TC 22/SC 39.

[CR17] Jasper, H. H. (1958). The ten-twenty electrode system of the international federation. *Electroencephalography and Clinical Neurophysiology,**10*, 370–375. 10590970

[CR18] Jensen, O., & Mazaheri, A. (2010). Shaping functional architecture by oscillatory alpha activity: Gating by inhibition. *Frontiers in Human Neuroscience,**4*, 186. 21119777 10.3389/fnhum.2010.00186 PMC2990626

[CR19] Kahneman, D. (1973). *Attention and effort*. Prentice-Hall.

[CR20] Käthner, I., Wriessnegger, S. C., Müller-Putz, G. R., Kübler, A., & Halder, S. (2014). Effects of mental workload and fatigue on the P300, alpha and theta band power during operation of an ERP (P300) brain–computer interface. *Biological Psychology,**102*, 118–129. 10.1016/j.biopsycho.2014.07.014 25088378 10.1016/j.biopsycho.2014.07.014

[CR21] Klimesch, W. (2012). Alpha-band oscillations, attention, and controlled access to stored information. *Trends in Cognitive Sciences,**16*(12), 606–617. 23141428 10.1016/j.tics.2012.10.007 PMC3507158

[CR22] Koo, T. K., & Li, M. Y. (2016). A guideline of selecting and reporting intraclass correlation coefficients for reliability research. *Journal of Chiropractic Medicine,**15*(2), 155–163. 27330520 10.1016/j.jcm.2016.02.012 PMC4913118

[CR23] Lee, H. B., Kim, J. S., Kim, Y. S., Baek, H. J., Ryu, M. S., & Park, K. S. (2007). The relationship between HRV parameters and stressful driving situation in the real road. In *6th International Special Topic Conference on Information Technology Applications in Biomedicine, 2007* (Tokyo). 10.1109/ITAB.2007.4407380

[CR24] Lenneman, J. K., & Backs, R. W. (2009). Cardiac autonomic control during simulated driving with a concurrent verbal working memory task. *Human Factors,**51*, 404–418. 10.1177/0018720809337716 19750801 10.1177/0018720809337716

[CR25] Lohani, M., Cooper, J. M., Erickson, G. G., Simmons, T. G., McDonnell, A. S., Carriero, A. E., Crabtree, K. W., & Strayer, D. L. (2021). No difference in arousal or cognitive demands between manual and partially automated driving: A multi-method on-road study. *Frontiers in Neuroscience,**15*, 577418. 10.3389/fnins.2021.577418 34177439 10.3389/fnins.2021.577418 PMC8222579

[CR26] Lohani, M., Cooper, J. M., Erickson, G., Simmons, T., McDonnell, A., Crabtree, K. W., & Strayer, D. L. (2020). Driver arousal and workload under partial vehicle automation: A pilot study. *Proceedings of the Human Factors Ergonomic Society.,**64*, 1955–1959. 10.1177/1071181320641471

[CR27] Lohani, M., Payne, B. R., & Strayer, D. L. (2019). A review of psychophysiological measures to assess cognitive states in real-world driving. *Frontiers in Human Neuroscience,**13*, 57. 10.3389/fnhum.2019.00057 30941023 10.3389/fnhum.2019.00057 PMC6434408

[CR28] Lopez-Calderon, J., & Luck, S. J. (2014). ERPLAB: An open-source toolbox for the analysis of event-related potentials. *Frontiers in Human Neuroscience,**8*, 213. 10.3389/fnhum.2014.00213 24782741 10.3389/fnhum.2014.00213 PMC3995046

[CR29] Luck, S. J. (2014). *An introduction to the event-related potential technique*. MIT press.

[CR30] Matthews, G., Reinerman-Jones, L. E., Barber, D. J., & Abich, J., IV. (2015). The psychometrics of mental workload: Multiple measures are sensitive but divergent. *Human Factors,**57*(1), 125–143. 25790574 10.1177/0018720814539505

[CR31] McDonald, R. P. (1999). Test theory: A unified treatment. Mahwah, N.J.: L. Erlbaum Associates.

[CR32] McDonnell, A. S., Simmons, T. G., Erickson, G. G., Lohani, M., Cooper, J. M., & Strayer, D. L. (2023). This is your brain on autopilot: Neural indices of driver workload and engagement during partial vehicle automation. *Human Factors,**65*(7), 1435–1450. 34414813 10.1177/00187208211039091 PMC10626989

[CR33] McGraw, K. O., & Wong, S. P. (1996). Forming inferences about some intraclass correlation coefficients. *Psychological Methods,**1*(1), 30. 10.1037/1082-989X.1.1.30 10.1037/1082-989X.1.1.30

[CR34] Mehler, B., Reimer, B., & Wang, Y. (2011). A comparison of heart rate and heart rate variability indices in distinguishing single-task driving and driving under secondary cognitive workload. In *Proceedings of the 6th International Driving Symposium on Human Factors in Driver Assessment, Training, and Vehicle Design: Driving Assessment, 2011* (Lake Tahoe, CA), 590–597. 10.17077/drivingassessment.1451

[CR35] Mehler, B., Reimer, B., & Coughlin, J. F. (2012). Sensitivity of physiological measures for detecting systematic variations in cognitive demand from a working memory task: An on-road study across three age groups. *Human Factors,**54*, 396–412. 10.1177/0018720812442086 22768642 10.1177/0018720812442086

[CR36] National Highway Traffic Safety Administration (2013). *Visual- Manual NHTSA Driver Distraction Guidelines for In-Vehicle Electronic Devices* (Federal Register Vol.78, No. 81). National Highway Traffic Safety Administration.

[CR37] National Transportation Safety Board. (2020). Collision between a sport utility vehicle operating with partial driving automation and a crash attenuator: Mountain View, California, March 23, 2018.

[CR38] Navon, D., & Gopher, D. (1979). On the economy of the human-processing system. *Psychological Review,**86*, 214–255. 10.1037/0033-295X.86.3.214

[CR39] Nunez, P. L., & Srinivasan, R. (2006). *Electric Fields of the Brain: The Neurophysics of EEG*. Oxford University Press.

[CR40] Reimer, B., Mehler, B., Coughlin, J. F., Godfrey, K. M., & Chuanzhong, T. (2009). An on-road assessment of the impact of cognitive workload on physiological arousal in young adult drivers. In *Proceedings of the 1st International Conference on Automotive User Interfaces and Interactive Vehicular Applications*, (ACM), 115–118. 10.1145/1620509.1620531

[CR41] Revelle, W. (2013). Using R and the psych package to find ω. Computer Software]. http://personality-project.org/r/psych/HowTo/omega.tutorial/omega.html #x1-150005.1.

[CR42] Revelle, W., & Condon, D. M. (2019). Reliability from α to ω: A tutorial. *Psychological Assessment,**31*(12), 1395. 31380696 10.1037/pas0000754

[CR43] Revelle, W., & Zinbarg, R. E. (2009). Coefficients alpha, beta, omega and the glb: Comments on Sijtsma. *Psychometrika,**74*(1), 145–154. 10.1007/s11336-008-9102-z 10.1007/s11336-008-9102-z

[CR44] RStudio Team. (2016). Rstudio: Integrated development environment for r [Computer software manual]. Boston, MA. Retrieved from http://www.rstudio.com/

[CR45] Ruscio, D., Bos, A. J., & Ciceri, M. R. (2017). Distraction or cognitive overload? Using modulations of the autonomic nervous system to discriminate the possible negative effects of advanced assistance system. *Accident Analysis and Prevention,**103*, 105–111. 10.1016/j.aap.2017.03.023 28399463 10.1016/j.aap.2017.03.023

[CR46] SAE (2016). Taxonomy and Definitions for Terms Related to Driving Automation Systems for on-Road Motor Vehicles (Surface Vehicle Recommended Practice: Superseding J3016 Jan 2014), *SAE International*. Available online at: https://www.sae.org/standards/content/j3016_201806/.

[CR47] Scerbo, M. (2007). Adaptive automation. *Neuroergonomics The Brain at Work*, 239252.

[CR48] Schmidt, E. A., Schrauf, M., Simon, M., Fritzsche, M., Buchner, A., & Kincses, W. E. (2009). Drivers’ misjudgement of vigilance state during prolonged monotonous daytime driving. *Accident Analysis & Prevention,**41*, 1087–1093. 10.1016/j.aap.2009.06.007 19664450 10.1016/j.aap.2009.06.007

[CR49] Shrout, P. E., & Fleiss, J. L. (1979). Intraclass correlations: Uses in assessing rater reliability. *Psychological Bulletin,**86*(2), 420. 10.1037/0033-2909.86.2.420 18839484 10.1037/0033-2909.86.2.420

[CR50] Shrout, P. E., & Lane, S. P. (2012). *Psychometrics*. Guilford Press.

[CR51] Statista (2023). Number of autonomous vehicles globally in 2022, with a forecast through 2030. https://www.statista.com/statistics/1230664/projected-number-autonomous-cars-worldwide/

[CR52] Strayer, D. L. (2015). Attention and driving. *The Handbook of Attention,**1*, 423–442.

[CR53] Task Force of the European Society of Cardiology. (1996). Heart rate variability, standards of measurement, physiological interpretation, and clinical use. *Circulation,**93*, 1043–1065. 10.1161/01.CIR.93.5.1043 8598068 10.1161/01.CIR.93.5.1043

[CR54] Taylor-Phillips, S., Jenkinson, D., Stinton, C., Kunar, M. A., Watson, D. G., Freeman, K., & Clarke, A. (2024). Fatigue and vigilance in medical experts detecting breast cancer. *Proceedings of the National Academy of Sciences,**121*(11), e2309576121. 10.1073/pnas.2309576121 PMC10945845 38437559

[CR55] Teo, T., & Fan, X. (2013). Coefficient alpha and beyond: Issues and alternatives for educational research. *The Asia-Pacific Education Researcher,**22*(2), 209–213. 10.1007/s40299-013-0075-z10.1007/s40299-013-0075-z 10.1007/s40299-013-0075-z10.1007/s40299-013-0075-z

[CR56] Tozman, T., Magdas, E. S., MacDougall, H. G., & Vollmeyer, R. (2015). Understanding the psychophysiology of flow: A driving simulator experiment to investigate the relationship between flow and heart rate variability. *Computers in Human Behavior,**52*, 408–418. 10.1016/j.chb.2015.06.023 10.1016/j.chb.2015.06.023

[CR57] Weir, J. P. (2005). Quantifying test-retest reliability using the intraclass correlation coefficient and the SEM. *The Journal of Strength and Conditioning Research,**19*(1), 231. 15705040 10.1519/15184.1

[CR58] Wickens, C. D. (1980). The structure of attentional resources. In R. S. Nickerson (Ed.), *Attention and performance VIII* (pp. 239–257). Erlbaum.

[CR59] Wickens, C. D. (1984). Processing resources in attention. In R. Parasuraman & R. Davies (Eds.), *Varieties of attention* (pp. 63–101). Academic Press.

[CR60] Young, R. A., Hsieh, L., & Seaman, S. (2013). The tactile detection response task: preliminary validation for measuring the attentional effects of cognitive load. In *Proceedings of the Seventh International Driving Symposium on Human Factors in Driver Assessment, Training and Vehicle Design*, New York, NY, 71–77. 10.17077/drivingassessment.1469

[CR61] Zander, T. O., Andreessen, L. M., Berg, A., Bleuel, M., Pawlitzki, J., Zawallich, L., et al. (2017). Evaluation of a dry EEG system for application of passive brain-computer interfaces in autonomous driving. *Frontiers in Human Neuroscience,**11*, 78. 10.3389/fnhum.2017.00078 28293184 10.3389/fnhum.2017.00078 PMC5329046

[CR62] Zhao, C., Zhao, M., Liu, J., & Zheng, C. (2012). Electroencephalogram and electrocardiograph assessment of mental fatigue in a driving simulator. *Accident Analysis and Prevention,**45*, 83–90. 10.1016/j.aap.2011.11.019 22269488 10.1016/j.aap.2011.11.019

[CR63] Zinbarg, R. E., Yovel, I., Revelle, W., & McDonald, R. P. (2006). Estimating generalizability to a latent variable common to all of a scale’s indicators: A comparison of estimators for ωh. *Applied Psychological Measurement,**30*(2), 121–144. 10.1177/0146621605278814 10.1177/0146621605278814

